# Clockor2: Inferring Global and Local Strict Molecular Clocks Using Root-to-Tip Regression

**DOI:** 10.1093/sysbio/syae003

**Published:** 2024-02-17

**Authors:** Leo A Featherstone, Andrew Rambaut, Sebastian Duchene, Wytamma Wirth

**Affiliations:** Peter Doherty Institute for Infection and Immunity, University of Melbourne, Melbourne, VIC 3010, Australia; Institute of Ecology and Evolution, University of Edinburgh, Edinburgh, UK; Peter Doherty Institute for Infection and Immunity, University of Melbourne, Melbourne, VIC 3010, Australia; Department of Computational Biology, Institut Pasteur, Paris, France; Peter Doherty Institute for Infection and Immunity, University of Melbourne, Melbourne, VIC 3010, Australia

**Keywords:** evolutionary rate heterogeneity, molecular clock, root-to-tip regression

## Abstract

Molecular sequence data from rapidly evolving organisms are often sampled at different points in time. Sampling times can then be used for molecular clock calibration. The root-to-tip (RTT) regression is an essential tool to assess the degree to which the data behave in a clock-like fashion. Here, we introduce Clockor2, a client-side web application for conducting RTT regression. Clockor2 allows users to quickly fit local and global molecular clocks, thus handling the increasing complexity of genomic datasets that sample beyond the assumption of homogeneous host populations. Clockor2 is efficient, handling trees of up to the order of $10^{4}$ tips, with significant speed increases compared with other RTT regression applications. Although clockor2 is written as a web application, all data processing happens on the client-side, meaning that data never leave the user’s computer. Clockor2 is freely available at https://clockor2.github.io/.

## Introduction

Phylodynamic analyses make use of genetic sequence data to understand the evolution, epidemiological, and ecological dynamics of a pathogen. Importantly, phyodynamics achieves its greatest value when generating insight about infectious disease dynamics beyond that offered by traditional epidemiological data. This frequently occurs at population interfaces, such as during transmission across host sub-populations, geographical boundaries, or host species. Despite the increased complexity of such datasets, the essential component to all phylodynamic modeling is the assumption of a molecular clock relating epidemiological and evolutionary timescales (Biek et al. 2015).

The simplest molecular clock model is the strict clock, which assumes a constant rate of substitution per unit time known as the “evolutionary rate” (Zuckerkandl and Pauling 1965). When the evolutionary rate is constant throughout a phylogenetic tree, the term *global* molecular clock is used. In contrast, a strict *local* clock refers to the situation where different substitution rates apply to different monophyletic groups within a tree (Ho and Duchêne 2014). The branches of local clocks are sometimes referred to as the “foreground” while the remaining branches are known as the “background,” such that there are foreground and background rates of evolution (Yoder and Yang 2000). The assumption of a local clock may, for example, correspond to sampling from different host populations, host species, or pathogen lineages (Worobey et al. 2014).

Several tools allow for the inference of strict molecular clocks via root-to-tip (RTT) regression, but none readily offers the ability to fit local clock models (Rambaut et al. 2016; Volz and Frost 2017; Hadfield et al. 2018; Sagulenko et al. 2018). Here, we introduce Clockor2, an RTT regression tool enabling rapid inference of global and local strict molecular clocks from phylogenetic trees where tips are annotated with sampling times and other relevant data. Clockor2 is bundled with an example from Dudas et al. (2018) where local clocks are fit to MERS-CoV samples from human and camel hosts, and another in the documentation from Porter et al. (2023) with SARS-CoV-2 samples from human and mink hosts.

Phylodynamic datasets are and will continue to grow in size and scope (Featherstone et al. 2022). For example, datasets of thousands to tens-of-thousands of samples have been used to understand the spread of SARS-CoV-2 at international scales, the emergence of variants of concern (VOC), and transmission between species (du Plessis et al. 2021; Hill et al. 2022; Nadeau et al. 2023; Porter et al. 2023). However, larger datasets are more likely to sample from distinct populations as a function of their size, making local clocks increasingly important. Currently, testing the fit of a local clock over alternative models, such as global or relaxed clocks, frequently requires intensive computational efforts using common Bayesian phylodynamic applications such as BEAST or RevBayes (Drummond and Rambaut 2007; Drummond et al. 2012; Höhna et al. 2016; Suchard et al. 2018; Bouckaert et al. 2019). Each generally requires hours to days of computation time. Clockor2 uniquely offers a scalable and accessible client-side web application for exploring the fit of local clocks, with results available in seconds to minutes to direct subsequent phylodynamic analysis.

**Figure 1:**
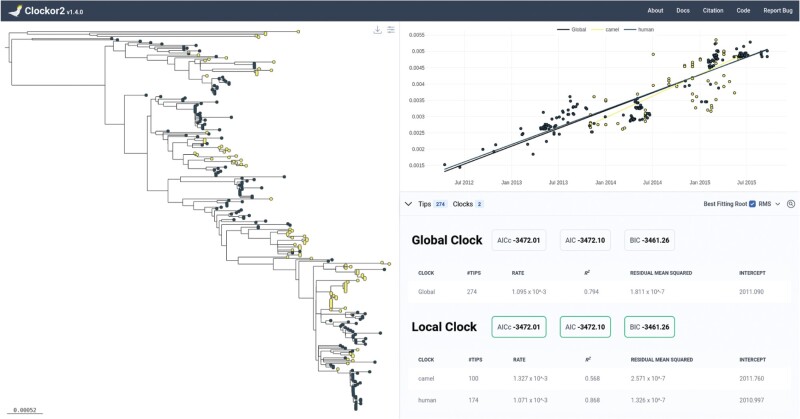
Clockor2 presents the tree alongside RTT regression data. Users can toggle between local and global clocks and alter the appearance of the tree. In this case, a tree of MERS‑CoV samples from Dudas et al. (2018) is presented with light coloured (yellow in online version) points corresponding to samples from camel hosts and dark coloured points (dark blue in online version) corresponding to human hosts. The solid dark line in the regression panel refers to the global clock where all samples are pooled.

Specifically, Clockor2 allows users to perform RTT regression for fitting global and local clocks (Fig. 1). The user begins by dropping or importing a rooted tree. Sampling dates and group identifiers can then be parsed from tip labels or separate files on input. Like other RTT regression applications, Clockor2 also allows users to infer the best fitting root based on the $R^{2}$ value or residual mean square (RMS) of the RTT regression. Both are key indicators of clock-like evolution (Drummond et al. 2003; Rambaut et al. 2016). It also offers users a local clock-search function to explore assumptions about the number of local clocks in a dataset as well as the ability to add a local clock interactively.

## Methods

### Documentation and Examples

The documentation for Clockor2 is available at https://clockor2.github.io/docs/. It includes demonstration of use with empirical datasets of MERS-CoV and SARS-CoV-2 from Dudas et al. (2018) and Porter et al. (2023). The MERS-CoV dataset can be loaded from the landing page and is also shown in Figure 1.

### General Model for Global and Local Strict Clocks

RTT regression consists in modeling the evolutionary rate as the slope of a linear regression of the distance from the root to each tip (RTT distance), typically in units of substitutions per site (s⁢u⁢b⁢s/s⁢i⁢t⁢e), against the sampling date of each tip (Drummond et al. 2003). If we denote the evolutionary rate as r (usually in units of s⁢u⁢b⁢s/s⁢i⁢t⁢e/t⁢i⁢m⁢e), RTT distance as d (usually in units of s⁢u⁢b⁢s/s⁢i⁢t⁢e), o as the intercept (interpreted as origin), and sampling times as t, then the model for a global strict clock takes the form:


d=r⁢t+o+ϵ

where ϵ is an error term.

Clockor2 uses a generalization of this model to accommodate local clocks. For a given tree with a set of tips T, we define local clocks as pertaining to *groups* of tips $g_{i}$ and a rate parameter for each ($r_{i}$). For a strict clock model with two local clocks, we then write


$$d=\left\{ \begin{array}{cc} r_{1}\,t+o_{1}+\epsilon ,\text{ if tip }\in g_{1} & \\ r_{2}\,t+o_{2}+\epsilon ,\text{ if tip }\in g_{2} & \end{array}\right.$$

**Figure 2:**
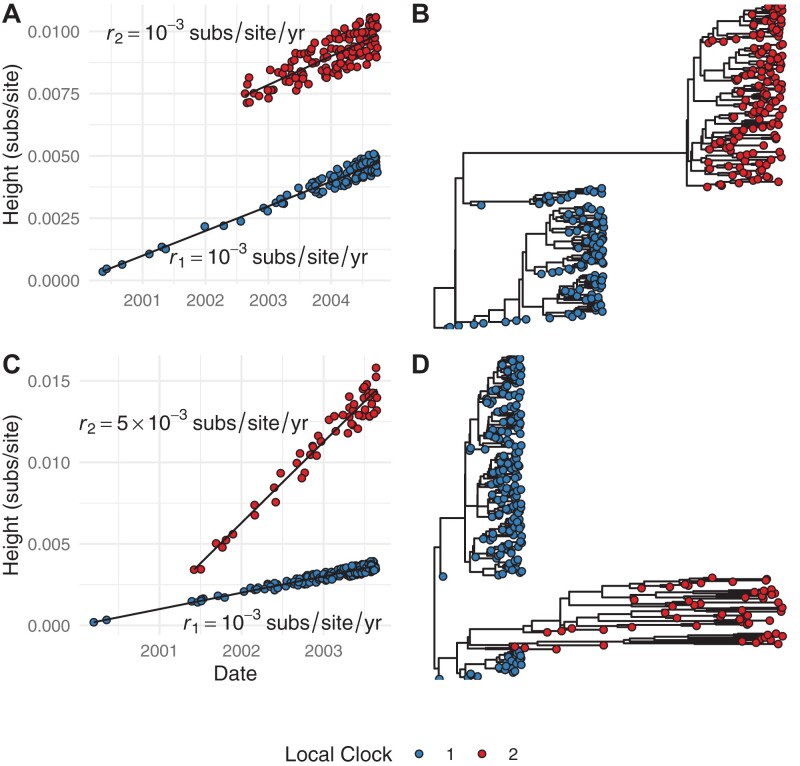
Simulated examples of how local clocks appear in trees and RTT regression data. a) RTT regression data for two local clocks with similar rates separated by a long branch. b) A tree characteristic of two similar local clock rates separated by a long branch. c) RTT regression data where two local clocks have different evolutionary rates. d) A tree characteristic of two local clocks with different rates.

We refer to *groups* instead of *clades* because while collections of tips belonging to one local clock necessarily share a common ancestor, they do not necessarily comprise a whole clade. This occurs when two or more local clocks are nested. The tips comprising the outer clock(s) then cannot comprise a whole clade if another local clock is nested within it. For example, local clock 1 shown in Figure 2b,d does not comprise a clade (i.e., is not monophyletic) because local clock 2 is nested within it.

This general model then captures the two key scenarios where local clocks may be appropriate. The first is where rates are similar between local clocks, but separated by a long branch (Fig. 2a,b). For example, in the evolution of VOCs in SARS-CoV-2 or due to temporally sparse sampling in the case of ancient *Yersisnia pestis* samples (Hill et al. 2022; Tay et al. 2022; Eaton et al. 2023). The second scenario is where rates differ between local clocks (Fig. 2c,d). For example, this can occur when a pathogen spreads in different host species, such as has been observed for SARS-CoV-2 in mink and human hosts (Porter et al. 2023).

For each group of tips defining a local clock, we independently conduct an RTT regression to estimate the evolutionary rate (slope). $R^{2}$ or RMS values are then an indication of clock-like behavior for each local clock. Clockor2 focuses on $R^{2}$ and RMS as indicators of clock-like evolution because they offer the most straightforward interpretation of clock-like evolution. $R^{2}$ values of 1 indicate perfect clock-like evolution, while values of 0 indicate a lack of a molecular clock. Likewise, lower RMS values indicate better fit of a strict clock.

Local clock and or global clock configurations can also be compared using an information criterion that combines the likelihood of each local clock’s RTT regression while penalizing the number of inferred parameters (three for each clock—slope, intercept, and variance). Clockor2 allows users to use either the Bayesian Information Cirterion (BIC), Aikake Information Criterion (AIC), or corrected Aikake Information Cirterion (AICc). We recommend using the BIC because it most heavily penalizes the addition of extra parameters, and local clocks in turn.

Derivations of the above information criteria for the local clock model are given in Supplementary material, Section 1. Briefly, these exploit the assumption of independent sampling to factor the likelihood across local clocks. Note, however, that this assumption is always flawed because samples necessarily share some ancestry by the assumption of a phylogenetic tree. In other words, ancestral branches are counted over many times in calculating the distance from root to tip for each sample (Duchêne et al. 2016). However, this is a limitation of the RTT regression approach generally, rather than of Clockor2 itself.

### Local Clock Search: An Exploratory Feature

Where it is hypothesized that a dataset contains local clocks, Clockor2 provides functionality to corroborate this hypothesis by performing a search for local clocks in the tree. Briefly, the algorithm takes a maximum number of clocks and a minimum number of tips (group size) for each local clock as input parameters. It then iterates through all combinations of internal nodes from which local clocks could descend to induce corresponding local clock configurations. Importantly, the clock search algorithm tests for a number of clocks up to and including the maximum number so that it may find more parsimonious configurations with fewer clocks. Configurations are compared using the information criteria outlined above. Again, we recommend the BIC as it penalizes additional parameters (i.e., additional local clocks) most heavily. See here for an animation (https://github.com/LeoFeatherstone/clockor2Paper  /  blob / main / figures  /  clockSearchEg2Clocks.gif) of the clock search algorithm.

The clock-search algorithm operates in polynomial time (see Supplementary material, Section 2). Efficiency is improved by reducing the maximum number of local clocks in the search, increasing the minimum group size, and contingent on the topology of the underlying tree. However, the former two parameters exert a far greater effect on efficiency than topology.

#### Clock-Search User Guidelines

We stress that this algorithm is intended as an *exploratory* feature of Clockor2, rather than a formal test. It has a strong tendency to over-parameterise and select higher numbers of local clocks, even where these all have congruent evolutionary rates (see Supplementary material, Section 3 and Fig. S2). Based on this, we only suggest using the clock search if there is a biological hypothesis about why there may be a particular number of clocks. In this case, users should test up to and including the hypothesized number of clocks, but not more, to avoid the high-likelihood of over-fitting. The clock search is intended to help corroborate hypotheses about numbers of local clocks in a dataset, and we urge users to formally test these hypotheses using more rigorous methods, such as a marginal likelihood comparison if they wish to report results (Tay et al. 2023; Drummond and Suchard 2010). The Phylostems software also provides a web-based platform to explore local temporal signal in trees (Doizy et al. 2023). We point users to documentation on the current limitations of the clock search (https://clockor2.github.io/docs/examples/sars-cov-2/).

### Finding the Best Fitting Root

Clockor2 selects the best fitting root based on the $R^{2}$ or RMS of a global clock model for the input tree. It seeks a root minimizing among-lineage rate variation, which is equivalent to maximizing temporal signal. It follows the same algorithm as implemented in TempEst (Rambaut et al. 2016), but makes use of parallelization to improve speed for larger trees. Briefly, the tree is rooted along the branch leading to each internal node or tip, an RTT regression is performed, and the root position along the branch leading to the highest $R^{2}$ or RMS is selected. When targeting $R^{2}$, Clockor2 optimizes the root position using the golden-section search algorithm (Kiefer 1953). There is an analytical solution for the RMS (see Supplementary material, Section 4).

The best fitting root is inferred using a single, global clock because it presents the most parsimonious model of the evolutionary rate for a given tree. The fit of more elaborate local clock models can then be compared with this using information criteria and/or comparing the $R^{2}$ or RMS values of each model. Clockor2 does not find the best fitting root for local clock models because the search space of best fitting roots and local clock configurations quickly becomes prohibitive and is possibly unidentifiable.

If a biologically informed root is available, such as with an outgroup, we suggest users retain it instead of the best fitting root. This is because the best fitting root essentially seeks to minimize among-lineage rate variation, which may contradict the biological reality.

### Dependencies

Clockor2 has three key dependencies for handling, and plotting trees and RTT data. Trees are handled and manipulated using the PhyloJS (https://www.npmjs.com/package/phylojs) library. Phylocanvas is used to visualize trees and plotly.js is used to plot RTT data (Abudahab et al. 2021; Plotly-Technologies-Inc. 2015).

## Results

### Efficiency

Clockor2 can process trees of up to the order of $10^{4}$ tips, and is thus fit for the expanding size and diversity of phylodynamic datasets. Finding the best fitting root makes use of parallelization to increase speed. Speedup is therefore proportional to the number of threads or cores available, in addition to the choice of browser and computer. For example, Clockor2 is faster than TempEst, v1.5.3, on a 2021 Macbook pro with 16 Gb of memory and 8 cores running Chrome, v113.0.5627.126 (Table 1). However, we found Clockor2 to be comparable or slower on other combinations of processor and browser, such as a Lenovo Thinkpad with an 11^th^ Gen Intel i7 processor running Firefox v118.0.2.

**Table 1:** Time in seconds taken to find the best fitting root for test trees of 100, 500, 1000, 5000, and 10,000 tips using Clockor2 and TempEst v1.5.3 on a 2021 Macbook with 16 Gb of memory and 8 cores running Chrome v113.0.5627.126. Times vary with computer and browser. In general, the relative efficiency of Clockor2 will increase with the number of cores. Using the residual mean squared as an optimization target is faster because there is an analytical solution.

	$R^{2}$		Residual mean squared	
Tips	Clockor2	TempEst	Clockor2	TempEst
100	0.313	0.760	0.129	0.050
500	1.370	2.400	0.502	1.500
1000	3.476	10.280	1.514	5.430
5000	78.013	272.290	24.370	122.000
10,000	306.821	1310.340	94.992	951.000
				

The user interface also remains responsive when working with large trees. This is in large part due to the use of WebGL in the tree and plotting components, which exploit GPU acceleration to render large and interactive trees and datasets through Phylocanvas and Plotly.js, respectively (Abudahab et al. 2021; Plotly-Technologies-Inc. 2015).

## Discussion

Clockor2 provides a flexible and scalable front-end web platform for RTT regression. Its extension to fitting local clocks allows it to accommodate the growing complexity of phylodyanmic datasets as genomic epidemiology plays a growing role in infectious disease surveillance.

As a front end application, Clockor2 is also highly accessible with no installation steps required, although users have option of saving the site to run locally. Wherever there is a browser, it is possible to conduct an RTT regression using Clockor2 with the data never leaving the user’s computer. This is particularly valuable where data sharing restrictions apply, such as for patient confidentiality.

### Future Directions

One future direction consists in finding the right information criterion to penalize the addition of local clocks in the clock search. This will help to transition the clock search from being an exploratory feature, to a formal test for finding local clocks. This may come in borrowing from the broader literature on continuously evolving traits (Khabbazian et al. 2016; Bastide et al. 2017). Non-parametric clustering approaches may also offer an alternative solution.

In the future, it will be possible to re-implement core functionality in increasingly popular and highly efficient programming languages that can compile to Web-Assembly format. For example, as the bioinformatics ecosystem in Rust continues to develop, it will be possible to further improve the efficiency of Clockor2 using packages such as Bio-Rust (Köster 2015).

## Data Availability

Clockor2 is available at https://clockor2.github.io/. All code required to replicate the simulation study and figures in the paper is available at https://github.com/LeoFeatherstone/clockor2Paper and https://datadryad.org / stash / dataset / doi:10.5061 / dryad.gxd2547sn. Thecode for Clockor2 is open source at https://github.com/clockor2/clockor2 and https://zenodo.org/records/10208495. No new empirical data are associated with this article.

## Supplementary Material

Data available from the Dryad Digital Repository: http://dx.doi.org/10.5061/dryad.gxd2547sn.
